# A Clinical Tool (CUE-tool) for Health Care Professionals to Assess the Usability and Quality of the Content of Medical Information Websites: Electronic Delphi Study

**DOI:** 10.2196/22668

**Published:** 2021-02-17

**Authors:** Leonie Klompstra, Maria Liljeroos, Johan Lundgren, Brynja Ingadottir

**Affiliations:** 1 Department of Health, Medicine and Caring Sciences Linköping University Norrköping Sweden; 2 Centre for Clinical Research Sörmland Uppsala University Eskilstuna Sweden; 3 Faculty of Nursing School of Health Sciences University of Iceland Reykjavik Iceland; 4 Landspitali University Hospital Reykjavik Iceland

**Keywords:** self-care, smartphone, internet, apps, websites, eDelphi

## Abstract

**Background:**

As patients are increasingly searching for information about their medical condition on the internet, there is a need for health professionals to be able to guide patients toward reliable and suitable information sources on the internet.

**Objective:**

The aim of the study was to develop a clinical tool for health care professionals to assess the usability and quality of the content of websites containing medical information that could be recommended to patients.

**Methods:**

A 3-round modified electronic Delphi (eDelphi) study was conducted with 20 health care professionals.

**Results:**

In round one of the eDelphi study, of the 68 items initially created, 41 items (29 on usability and 12 on content) were rated as important or very important by more than half of the panel and thus selected for further evaluation in round two. In round two, of the 41 items chosen from round 1, 19 were selected (9 on usability and 10 on content) as important or very important by more than half of the panel for further evaluation. As a result of round three, 2 items were combined as a single item, leaving the instrument with 18 items in total (8 on usability and 10 on content). The tool is freely accessible online.

**Conclusions:**

The CUE-tool can be used to (1) evaluate the usability and reliability of the content of websites before recommending them to patients as a good information source; (2) identify websites that do not have reliable content or may be difficult for patients to use; (3) develop quality websites by using the criteria in the CUE-tool; and (4) identify different qualities between different websites.

## Introduction

### Background

Patients are increasingly searching for information about their medical condition on the internet. In high-income countries 75% of the population reported that they search the internet for health information [1,2]. Using the internet as a source for different types of health-related information (eg, reading about medications, reading about other persons’ health experiences, watching health-related videos, and signing up for different health email updates) is more common among patients with a chronic illness compared with people without a chronic illness [2]. Health-related information on the internet is gradually replacing health professionals as important sources of reliable and independent information regarding health and treatment. There are advantages related to online patient education. For example, patients with chronic diseases reported, after seeking information from disease-specific websites, that they were taking their medications more regularly and adhering to treatment to a greater extent [3]. However, when patients lack guidance, there is a risk that their condition becomes worsened by, for example, waiting for too long seeking health care with symptoms of deterioration [4].

Although websites and smartphone apps are available with reliable information, there are also numerous examples of low-quality information regarding health and medical problems accessible through the internet [5]. Furthermore, the information could be very reliable (eg, peer-reviewed open access articles) but less suitable or understandable for the average patient.

Self-care (a rational process involving purposeful choices and behaviors, reflecting knowledge and thought [6]) is essential in the management of most illnesses, and knowledge about own health condition has been identified as an important prerequisite for successful self-care. To gain knowledge, the patient needs access to reliable and understandable information [7]. In order to gain knowledge, whether from the internet or other sources, the patient needs to be able to both read and understand the information [8]. As the information available on the internet is excessive, patients increasingly turn to health care for help with choosing reliable websites [9]. Besides, the information available on websites is not always of acceptable quality or usable for the patient. Therefore, there is a need for health professionals to be able to guide patients toward reliable and suitable information sources on the internet.

An important result of a comprehensive website evaluation [10] was the need for a practical, easy-to-use tool to evaluate websites. There are a number of different tools to evaluate websites; however, these tools seldom consider both the reliability and appropriateness of the information as well as the readability/comprehensibility of the information at the same time.

### Purpose

In this study, we aimed to develop a clinical tool for health care professionals to assess the usability and quality of the content of websites containing medical information that could be recommended to patients.

## Methods

### Electronic Delphi Study

**Figure 1:**
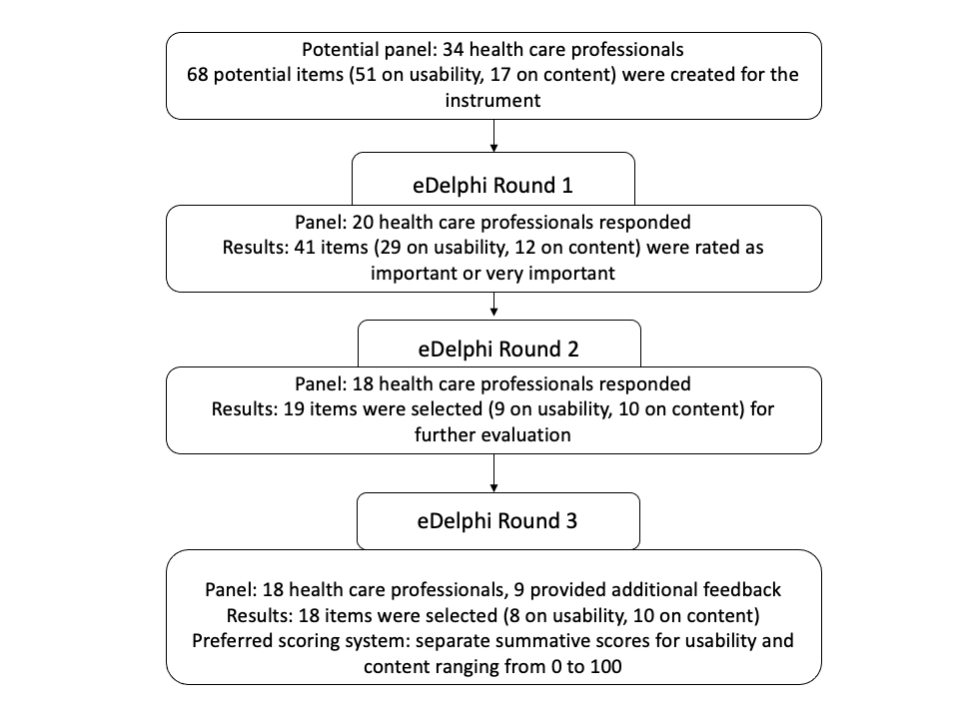
A 3-round eDelphi study for developing the CUE-tool. eDelphi: electronic Delphi.

For the development of a tool to assess the quality of websites containing health-related medical information for patients, a 3-round modified electronic Delphi (eDelphi) study was conducted (Figure 1). An eDelphi study is a structured process distributing a series of questionnaires during several rounds to gather information and set priorities or gain consensus regarding a specific issue [11,12]. The eDelphi technique allows the inclusion of a large number of individuals across diverse geographical locations without physically meeting them. The eDelphi technique is often conducted via online web surveys, offering a number of advantages, as they are quick to set up, relatively low cost, and provide high level of data security [13]. Systematic feedback, structured information flow, and iteration and anonymity are the main characteristics of an eDelphi technique. Systematic feedback of experts’ responses takes place in-between rounds by informing individual experts about the group opinions. Iteration takes place by presenting feedback via a certain number of rounds [12].

### Procedures and Participants

The panel consisted of health care professionals, selected based on their publications within patient education or that they were members of the CESAR (Collaboration and Exchange in Swedish cardiovascular caring Academic Research) network, a professional research network in Sweden. We aimed for a multidisciplinary panel of health care professionals, with diversity in gender and from variety of countries. The health care professionals that participated in the first round were also approached for the second and third rounds. Study data were collected and managed using REDCap electronic data capture tools hosted at the University of Iceland [14].

Before round one of the study’s eDelphi, items on usability and content were created based on available literature on website evaluation and the general information needs of patients. The theoretical perspective of empowering patient education [15] guided the development of the items in the content part of the tool. In empowering patient education, the emphasis is on patients’ knowledge expectations and the knowledge patients receive. The more patient expectations of knowledge are met with the received knowledge, the more possibilities there are for empowerment and self-management [15].

A total of 68 items were created for the first round, of which 58 were based on the available literature on tools for evaluation of websites and 10 on content from the multidimensional empowering patient education [15].

The panel was asked to rate the importance of each item on a 4-point scale (response options from 1 [Very important] to 4 [Not important at all]) and invited to comment on each item.

Items were selected for round two if more than half of the participants rated them as important or very important and the same method was applied for round three. In round three the panel was asked to answer 5 additional questions on the developed tool:

1. Are there any items missing?

2. Are there any items unclear?

3. Please look at the examples given in the tool. Do you agree on the examples or do you have suggestions for better examples?

4. Please give your thoughts about the end product of the tool.

5. Do you have a suggestion on a name of the instrument that is easy to use and reflects the area of use?

After the development of the tool, it was translated from English to Swedish, Icelandic, and Dutch and its face feasibility tested by nurses and allied professionals at the EuroHeartCare Conference 2019, Icelandic Nurses’ Association Conference Hjúkrun 2019, Icelandic nursing students, and in 2 primary care centers and 1 in-hospital heart failure clinic in Sweden. These health care professionals could choose any website including patient information, in any language, and they were asked if the aim of the development of the CUE-tool was met and their opinion about the usability of the scale. All of the health care professionals agreed that the aim was met, and they were positive about using the tool to evaluate websites. No adjustments were needed.

## Results

In total, 20 out of 34 health care professionals, from 5 countries, responded to the invitation to participate in the study in round one and 18 of them responded in rounds two and three. The panel came from Sweden, the Netherlands, Iceland, Norway, and Finland. The panel members’ age ranged from 30 to 69 (mean 46 [SD 11]) years and 15/20 were female (75%). Among the panel members, 13 had a doctorate, 4 had a master’s degree, and 1 had a bachelor’s degree (Table 1).

**Table 1:** Electronic Delphi (eDelphi) panel demographics.

Demographics		Round 1 (N=20)
Female, n (%)		15 (75)
Age (years), mean (SD)		46 (11)
**Education, n (%)**		
	Doctorate	13 (65)
	Master’s degree	4 (20)
	Bachelor’s degree	1 (5)
	Missing	2 (10)
Experience in patient education (years), mean (SD)		15 (10)
**Main area of work role, n (%)**		
	Clinical	1 (5)
	Research	12 (60)
	Clinical and research	6 (30)
	Other	1 (5)

In round one of the eDelphi study, of the 68 items initially created, 41 (29 on usability and 12 on content) were rated as important or very important by more than half of the panel and selected for further evaluation in round two (Figure 1).

In round two, of the 41 items chosen from round 1, 19 were selected (9 on usability and 10 on content) as important or very important by more than half of the panel, and thus these items were considered for further evaluation.

As a result of round three, 2 items were put together as a single item, leaving the instrument with 18 items in total (Multimedia Appendix 1). The panel did not miss any items in the tool, and all the items were clear. Examples given in the instrument to clarify items were also agreed on.

As an end product, the panel preferred a scoring system with 2 separate summative scores, 1 for usability and 1 for content, ranging from 0 to 100. From the suggestions made by the panel on a name for the tool, the authors chose the *CUE-tool* as an acronym for “The Credible and Usable Evaluation of patient education tool for web-sites.”

The nurses and allied professionals (N = 100) who tested the CUE-tool (on paper) when evaluating websites for patient educational purposes had no problems using it. All items were clear and the tool was seen as an addition to practice. To have the tool online with a summative scoring system was seen as an asset. The tool is freely accessible online [16] and a copy of the tool is also presented as Multimedia Appendix 1. A summative scoring and reliability assessment will be performed in the future.

## Discussion

Because patients are increasingly searching for information about their medical condition online, it was important to develop a tool for health care professionals so they can advise patients on suitable and reliable websites from which they can seek disease-related information.

The CUE-tool is an easy-to-use website evaluation tool that helps the user to evaluate both the website’s usability and quality of its content. A recent review [17] evaluated patients’ preferences for the design features of an effective online education website and found that the information should be patient tailored, interactive, and the content credible and clearly presented. Patients also found multimedia and high interpretability to be essential design features of online patient education websites for chronic disease management. All these features are assessed when using the CUE-tool.

The composition of the eDelphi panel may have affected the results of this study and the development of the tools because the majority were researchers, although of different backgrounds (age and profession). However, most of them had previous work experience as clinical nurses at hospitals, in both wards and outpatient clinics. Although we used patient knowledge expectations while developing the content items in the CUE-tool by using the theoretical perspective of empowering patient education [15], we did not include patients in the eDelphi panel. This decision was made because the tool was designed to be used as a clinical tool to assist health care professionals in finding websites that could be recommended to patients. Although the CUE-tool is developed to be used as a clinical tool, it is also useful outside of health care, for example, in patient organizations or in developing websites for patient education.

The use of empowering patient education [15] as the theoretical foundation of the content items and performing an eDelphi study to develop the CUE-tool may provide specific future possibilities of its applications in (nursing) research and education besides its clinical usefulness. For example, the CUE-tool can be used to (1) evaluate the usability and reliability of the content of websites before recommending them to patients as a good information source; (2) identify websites that do not have reliable content or may be difficult for patients to use; (3) develop quality websites using the criteria in the CUE-tool; (4) identify different qualities between different websites. Accurate and detailed assessments of available websites providing health information can be a valuable resource in teaching strategies to increase knowledge and self-care of patients. Accordingly, health care professionals can create new teaching interventions and revise curricula based on reliable websites identified in these assessments. For further validation, we will include patients’ perspectives and the reliability of the scoring system of the CUE-tool will be assessed in future research.

## Appendix

Multimedia Appendix 1 The CUE-tool: The Credible and Usable Evaluation of patient education tool for websites.

## Abbreviations

CESAR: Collaboration and Exchange in Swedish cardiovascular caring Academic Research
CUE-tool: The Credible and Usable Evaluation of patient education tool for websites
