# Supplementary material for: A Clinical Tool (CUE-tool) for Health Care Professionals to Assess the Usability and Quality of the Content of Medical Information Websites: Electronic Delphi Study
Source: J Med Internet Res. 2021 Feb 17;23(2):e22668. doi: 10.2196/22668 (PMC7929737; doi:10.2196/22668)
Supplement: Multimedia Appendix 1 [file jmir_v23i2e22668_app1.pdf]

The CUE Tool

The Credible and Usable Evaluation of patient education tool for websites

The CUE-Tool is designed as an evaluation tool to evaluate both usability and content of websites containing medical information that could be recommended to patients.

USABILITY

Please respond to the following questions by placing a “X” in the appropriate column that corresponds to your response

		YES	NO
1.	The lay-out and organization of the website is simple (e.g., not a lot of modules, no pop-up windows)		

		YES	NO
2.	The website has a consistent lay-out (e.g., standard page design and the same symbols and icons throughout; the same set of navigation buttons in the same place on each page to move from one web page or section of the website to another; labels each page in the same location with the name of the web site)		

		YES	NO
3.	Most sentences (more than 80%) are simple with no embedded information (not long, multiple phrases in a sentence)		

		YES	NO
4.	Text is stated in a positive/encouraging tone (example: “Don’t forget to take your medicine,” deliver the message positively: “Remember to take your medicine”)		

		YES	NO
5.	Focus in the text is on solutions patients can take (not just the problem)		

		YES	NO
6.	Examples are given to explain or clarify difficult words, concepts or category words (if they occur) most of the time (for example pregnancy test is negative, which means you are not pregnant)		

		YES	NO	N/A
7.	If illustrations are used, they are simple, likely to be familiar to/easily recognized by viewers and relevant to related message			

		YES	NO	N/A
8.	If videos or audio fragments are used, they are simple, likely to be familiar to/easily recognized by viewers and relevant to related message			

Please cite as: Klompstra L, Liljeroos M, Lundgren J, Ingadottir B. A Clinical Tool (CUE-tool) for Health Care Professionals to Assess the Usability and Quality of the Content of Medical Information Websites: Electronic Delphi Study. Journal of Medical Internet Research. 24/12/2020:22668. DOI: 10.2196/22668

CC BY

The CUE Tool

The Credible and Usable Evaluation of patient education tool for websites

CONTENT

Please respond to the following questions by placing a "X" in the appropriate column that corresponds to your response

1.	The content of the website is credible/reliable and based on:	YES	NO
a.	Guidelines		
b.	A theory		
c.	A model		
d.	Other:		

		YES	NO
2.	The owner of the website is mentioned		

		YES	NO
3.	The website is updated in the last year		

4.	The content includes information on the disease:	YES	NO
a.	Definition		
b.	Risk factors		
c.	Causes		
d.	Signs and symptoms		
e.	The consequences of the disease if not treated		

5.	The content includes information on the treatment of the disease:	YES	NO	N/A
a.	Medication			
b.	Surgery			
c.	Device implantation			
d.	Rehabilitation			
e.	Possible symptoms/side effects of the treatment			
f.	Reaction to symptoms/side effects of treatment			
g.	Possible complications of treatment			
h.	Response to complications to treatment			
i.	Complementary care or alternative therapy			
j.	Other:			

6.	The content includes information on how the disease/treatment may affect daily life, such as:	YES	NO
a.	Diet		
b.	Physical activity (e.g., exercise, mobility, exercise capacity, daily activities such as work, household chores or hobbies)		
c.	Sleep and rest		
d.	Sex		
e.	Hygiene (e.g., showering, bathing, toileting)		
f.	Other:		

Please cite as: Klompstra L, Liljeroos M, Lundgren J, Ingadottir B. A Clinical Tool (CUE-tool) for Health Care Professionals to Assess the Usability and Quality of the Content of Medical Information Websites: Electronic Delphi Study. Journal of Medical Internet Research. 24/12/2020:22668. DOI: 10.2196/22668

7.	The content includes information on how the disease/treatment may affect the patient psychologically such as:	YES	NO	N/A
a.	Common psychological responses to the disease and its treatment			
b.	How previous experience can be used when coping with the disease/treatment			

8.	The content includes information or a website link on the rights and duties of the patient:	YES	NO
a.	On shared decision making in treatment (e.g., right to a second opinion, right to refuse/decline treatment)		
b.	On how the patient can participate in own care (e.g. help with preparing appointments, prepare questions to ask health care professionals, self-care and adherence to treatment)		
c.	On what the patient can expect from health care professionals (information on ethical guidelines in treatment, confidentiality)		

9.	The content includes information or a website link on available social support, such as:	YES	NO
a.	How carers (anyone involved in the care of the patient: family, friends, neighbours) can be involved/contribute to the self-care and treatment of the disease		
b.	How the disease/treatment can affect carers		
c.	Patient organizations		
d.	Helplines		
e.	Private agents		
f.	Primary health care centres		
g.	Other:		

10.	The content includes information or a website link about the out-of-pocket expenditure a patient can expect as a result of the disease and its treatment such as cost of:	YES	NO	N/A
a.	Medication			
b.	Hospital appointments			
c.	Rehabilitation			
d.	Aids			
e.	Diet			
f.	Other:			

Please cite as: Klompstra L, Liljeroos M, Lundgren J, Ingadottir B. A Clinical Tool (CUE-tool) for Health Care Professionals to Assess the Usability and Quality of the Content of Medical Information Websites: Electronic Delphi Study. Journal of Medical Internet Research. 24/12/2020:22668. DOI: 10.2196/22668
